# Development and Testing of a Dual Accelerometer Vector Sensor for AUV Acoustic Surveys †

**DOI:** 10.3390/s17061328

**Published:** 2017-06-08

**Authors:** Agni Mantouka, Paulo Felisberto, Paulo Santos, Friedrich Zabel, Mário Saleiro, Sérgio M. Jesus, Luís Sebastião

**Affiliations:** 1 Laboratory for Robotics and Engineering Systems, University of Algarve, 8005-139 Faro, Portugal; pfelis@ualg.pt (P.F.); pjsantos@ualg.pt (P.S.); masaleiro@ualg.pt (M.S.); sjesus@ualg.pt (S.M.J.); 2 Marsensing Lda, 8005-139 Faro, Portugal; fredz@marsensing.com; 3 Instituto Superior Téchico, Institute for Systems and Robotics,1049-001 Lisbon, Portugal; l.sebastiao@tecnico.ulisboa.pt

**Keywords:** vector sensors, AUV, spatial filtering

## Abstract

This paper presents the design, manufacturing and testing of a Dual Accelerometer Vector Sensor (DAVS). The device was built within the activities of the WiMUST project, supported under the Horizon 2020 Framework Programme, which aims to improve the efficiency of the methodologies used to perform geophysical acoustic surveys at sea by the use of Autonomous Underwater Vehicles (AUVs). The DAVS has the potential to contribute to this aim in various ways, for example, owing to its spatial filtering capability, it may reduce the amount of post processing by discriminating the bottom from the surface reflections. Additionally, its compact size allows easier integration with AUVs and hence facilitates the vehicle manoeuvrability compared to the classical towed arrays. The present paper is focused on results related to acoustic wave azimuth estimation as an example of its spatial filtering capabilities. The DAVS device consists of two tri-axial accelerometers and one hydrophone moulded in one unit. Sensitivity and directionality of these three sensors were measured in a tank, whilst the direction estimation capabilities of the accelerometers paired with the hydrophone, forming a vector sensor, were evaluated on a Medusa Class AUV, which was sailing around a deployed sound source. Results of these measurements are presented in this paper.

## 1. Introduction

Acoustic vector sensors are relatively compact sensors with spatial filtering capabilities. Usually, they measure acoustic pressure and particle velocity and these signals are combined to produce an intensity estimation. After signal processing, the resulting beam is directional. Various signal processing schemes are encountered in the literature; for example, the particle velocity can be measured directly or as a derived value from acceleration or pressure differential, see [1], for the underlying theory. Applications of vector sensors include target tracking [2,3], detection and estimation of Direction of Arrival (DOA) of sound sources [4,5,6], underwater communication [7,8] and geo-acoustic inversion [9,10].

An important area of application for vector sensors is geo-acoustic surveys, where traditionally they are deployed on the earth surface or laid with cables on the seafloor. Owing to their directionality, they can distinguish between vertical and horizontal earth motions and hence they are used to record multicomponent seismic data. In sea surveys in particular, water-bottom cables with such sensors have been used for the attenuation of water-column reverberations [11]. In recent years, vector sensors have been used on towed streamers for the elimination of surface reflections (ghosts) [12], however details of these developments have limited publicity as they contain commercially sensitive information. A further advancement in marine geo-acoustic surveys is the replacement of the ship-towed streamers with sensors either towed or carried by Autonomous Underwater Vehicles (AUV). Within this area, to the best of the author’s knowledge, there are no dual vector sensors customized for this application that can be attached on AUVs without major integration procedures. The vector sensor device, which is described here, has two closely spaced accelerometers and a hydrophone, so that different signal processing schemes can be applied [1], see also Section 2.1.

The WiMUST (Widely scalable Mobile Underwater Sonar Technology) project [13], which is funded by the European Union under the Horizon 2020 Framework Programme, aims at expanding the functionalities of the current cooperative marine robotic systems, in order to enable deployment of distributed acoustic arrays for geophysical surveying in a setup composed of a ship towing a source and a receiving array (streamer) towed by AUV. These arrays consist of pressure sensors and a dedicated Dual Accelerometer Vector Sensor (DAVS), which will be mounted on one of the AUVs to demonstrate its advantages in this scenario. For example, it is expected that the DAVS will be able to distinguish between the sea-bottom reflections from surface and direct path signal, thereby eliminating this process from the standard towed array post-processing flow. Prior to this, the DAVS was tested in a simpler acoustic signal discrimination scenario, which is described here. The text describes the DAVS, a vector sensor with commercially available components, that can be attached to different platforms. Then, some of its acoustic characteristics are discussed, based on calibration measurements. Additionally, some preliminary experimental results, from an acoustic survey scenario, are shown as part of preparations that took place before the WiMUST trials.

Section 2 discusses the DAVS system and its calibration. The mechanical and electronic characteristics of the device are discussed in Section 2.1. Section 2.2 describes the acoustic characteristics, i.e., sensitivity and directivity, of the sensor elements as measured in a tank and Section 2.3 describes the conditions for the experiments with AUVs. Section 3 presents preliminary results with the DAVS device mounted on an AUV.

## 2. Materials and Methods

The DAVS device was built predominantly with off-the-self components, with the intention of being a low-cost sensor independently deployable from different platforms. To be flexible in use, the device can be powered autonomously for 20 h and can record and store data up to 128 gigabytes (GB). Moreover, the application of direction finding algorithms with a vector sensor requires knowledge of the sensor orientation relative to the ambient environment; for this reason, the device is equipped with motion sensors. In addition, knowledge of the individual sensor acoustic response within the overall construction is required in order to combine results from different sensors. The following paragraphs give details on these design aspects.

### 2.1. DAVS Design and Description

**Figure 1:**
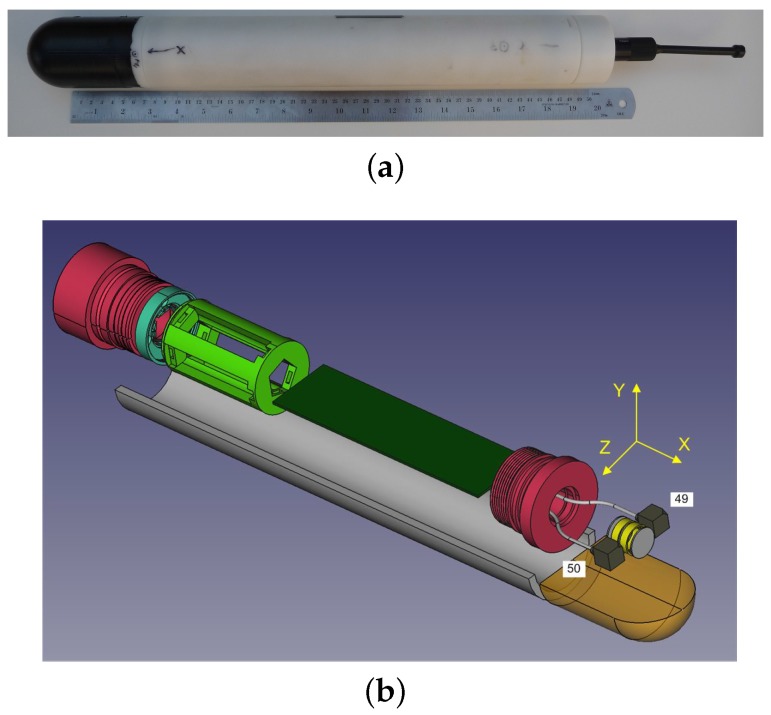
(**a**) Photo of Dual Accelerometer Vector Sensor (DAVS) and (**b**) exploded view of the DAVS in 3D solid modelling, showing the Delrin container (white half tube), the acoustically sensitive part (dark yellow), the two accelerometers (grey blocks), the hydrophone (yellow cylinder), the threaded caps (pink), the electronics (dark green block) and the battery pack (light green). The figure shows the coordinate convention of the device, which coincides with the three sensing dimensions of the accelerometers.

A photo of the DAVS is shown in Figure 1a, where the two main parts can be seen. The one is the acoustically sensitive part (black nose) which contains the acoustic sensors and the other part is a tube made of Delrin, which houses the electronics, acquisition system, batteries and motion sensor. The total length of the device is 525 mm and its diameter is 65 mm.

The device’s acoustic sensor is one in-house built, end-cupped cylindrical hydrophone made of PZT piezoelectric material and two tri-axial accelerometers acquired from PCB Piezotronics, model number 356A17. Throughout this paper, the naming of these accelerometers is the last two numbers of their serial number: 49 and 50. The numbering of the accelerometers with respect to their position in the device and the coordinate convention are shown in Figure 1b. Although in principle a single tri-axial accelerometer would be sufficient for the generic purpose of the DAVS, the double accelerometer design was motivated by the possibility of applying direction finding algorithms using particle velocity and differential acoustic particle measurements, which require two accelerometers, as explained in [1], and result in different beam widths. Additionally, the second accelerometer serves as a back-up in case of failure. The adopted configuration, with two closely measuring devices with a single shared pressure sensor is a compromise between the requirements above and the compactness of the whole device aimed to be carried by autonomous platforms.

Figure 1b shows an exploded view of a three-dimensional CAD model of the device. In this figure, we discern, in the acoustically sensitive part (in dark yellow) which represents the polyurethane mould, the DAVS sensing elements: two accelerometers (grey blocks) either side of the hydrophone (yellow cylindrical component). The sensors are moulded together with a threaded cap (in pink), which is screwed to the cylinder which contains the electronics (here represented as a dark green block). The cylinder is closed with another threaded cap (in pink) and contains the battery pack, shown in light green colour.

The acquisition system of the DAVS is a digital platform for the acoustic sensors and a non-acoustic motion sensor. Its electronic components include a micro-controller, an analogue multi-channel simultaneous acquisition system, data storage on a removable flash device, real-time clock, non-acoustic positioning sensors for pitch, roll and heading, power management and an external communications port. Table 1 gives an overview of the DAVS system design characteristics. The device can operate autonomously on batteries for 20 h and stores data in a microSD card. Alternatively, it can be powered externally to a 24 V DC power supply, streaming data via Ethernet.

There are two amplification stages. At the front end of the analogue signal, there is a 6 dB gain pre-amplifier with one pole high pass filter at 120 Hz for attenuation of low frequency vibrations originating from device motion. This first stage pre-amplifier has been designed to allow for a maximum input voltage of 10 Vpp, which for a hydrophone sensing element with a sensitivity of −195 dB re V/μ Pa permits a maximum input SPL of 209 dB re μ Pa. This is followed by a PGA with variable gain, which allows the user to select the second stage amplification according to the application. The SNR of the analogue-to-digital converter is 106 dB when operating at a sampling rate of 10,547 Hz and can go up to 110 dB when operating at a sampling rate of 52,734 Hz. Using a 24 bit sigma-delta analogue-to-digital converter (ADC), the flat passband frequency response for this acquisition system is 4.8 kHz and 23.9 kHz for the sampling rates of 10,547 Hz and 52,734 Hz respectively. This analogue-to-digital converter samples initially at 27 MHz and therefore owing to this oversampling technology an antialiasing filter is not needed. All channels, i.e., the three channels from each accelerometer and the channel from the hydrophone are recorded simultaneously.

Table 1 refers to the nominal characteristics of the device. The discussion in the following paragraphs of this paper is limited to the attained performance in a shallow water environment and a narrower frequency band as explained below.

### 2.2. DAVS Calibration

Prior to applying a direction finding algorithm to signals received by the DAVS device, it was necessary to check the performance of the sensing elements in the moulded unit. The frequency range of interest for the in situ experiments that are discussed in this paper is 1 kHz–2 kHz. However, results at 1 kHz were not trustworthy due to the difficulty in achieving free-field in the calibration tank at this frequency, therefore only the results at 2 kHz are shown here.

**Figure 2:**
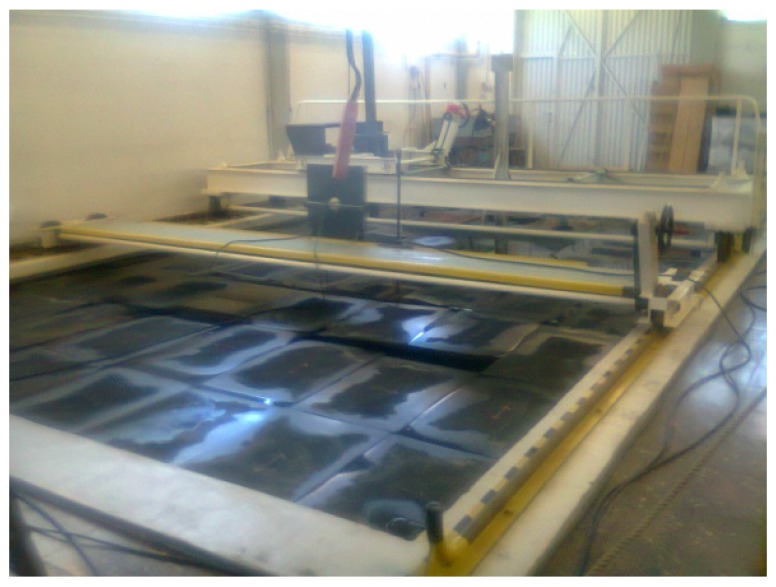
Photo of the Arsenal tank covered with reflection reducing panels, showing the two carriage positioning systems.

The tests took place in the tank Arsenal do Alfeite at the naval base in Lisbon. The tank has dimensions 8 m × 5 m × 5 m (length × width × depth) and is covered with wedged panels at all sides including the top surface in order to reduce reflections, as shown in Figure 2. The tank is equipped with two carriage systems for positioning the acoustic devices. For the calibration measurements, the double extensional transducer (‘dogbone’) model SX05 from Sensor Technology Ltd was used as the source.

The DAVS was rigidly mounted on a vertical cylinder and the source was placed in front of it at 2 m distance emitting tone bursts of 20 cycles; both devices were submerged at a depth of 2.5 m. With these settings, the received signals were ‘clean’, free from interferences, and achieved steady-state response for four cycles; the sensitivity was found by taking the root mean square value over this integral number of cycles, see annex C of [14]. The hydrophone sensitivity was measured by averaging these four cycles from twenty pulses, using the method ‘calibration by comparison’, as described in [15]. A calibrated Reson hydrophone TC4033, with sensitivity −202 dB re V/μ Pa up to 9 kHz, served as the reference hydrophone for these measurements, which was suspended by its cable for minimum disturbance of the acoustic field in the tank next to the DAVS. The DAVS hydrophone sensitivity was measured −196 ± 2 dB re V/μ Pa at 2 kHz.

Calibration standards do not prescribe procedures for accelerometer calibration in water. To estimate the acceleration sensitivity ($M_{a}$), first the equivalent pressure sensitivity ($M_{p}$) was estimated from the Reson hydrophone and then using the Relation 1, it was converted to acceleration sensitivity (see [16]), where ρ, *c* and ω are the water density, sound speed and the acoustic frequency respectively.

(1) $$M_{a}=\frac{\rho cM_{p}}{\omega }$$

At 2 kHz, the maximum acceleration sensitivity for both accelerometers on the *Z* and *Y* axis was found to be 24 ± 1 mV/m/s${}^{2}$. As expected, this value is lower than the corresponding value in air as quoted by the manufacturer (see Table 1). This is attributed to the water loading, which is the dominant effect when the accelerometer operates below resonance, as in this case.

**Figure 3:**
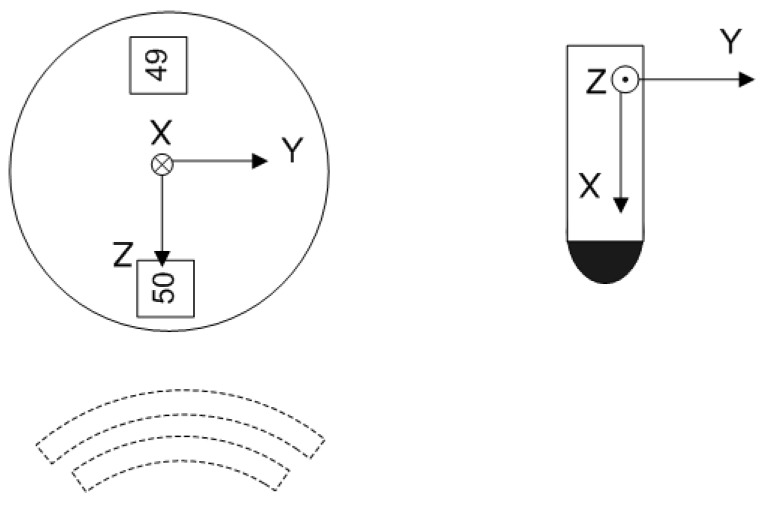
DAVS orientation at the starting position (0${}^{\circ }$) for directivity measurements; left top view and right side view of the device’s coordinate system. The *X* direction is pointing to the bottom of the tank.

Using the same sound source, i.e., the dogbone, the directional response of the hydrophone and the accelerometers were measured in the tank by mounting rigidly the DAVS device vertically on a rotating table with the orientation shown in Figure 3. By definition, directivity measurements require rotation of the device around its axis, which was possible only with this mounting arrangement, and therefore for the X-component the directional response was not measured. The rotation was continuous for the whole 360${}^{\circ }$ rotation. Additionally, these measurements require the measurements to be ‘in the far field, or region of the acoustic field for which the transducer response function varies inversely with range [17]’. Measurements with this setup and the hydrophone at 2 and 2.5 m from the source show that this condition was fulfilled within 0.5 dB error. The directional response pattern is presented in the form of a two-dimensional polar graph. The response is plotted in decibels for each angle and the value was normalised by the maximum. At the angle of 0${}^{\circ }$ of the directivity polar plots, the source was insonifying the accelerometer 50 first, as shown in Figure 3.

**Figure 4:**
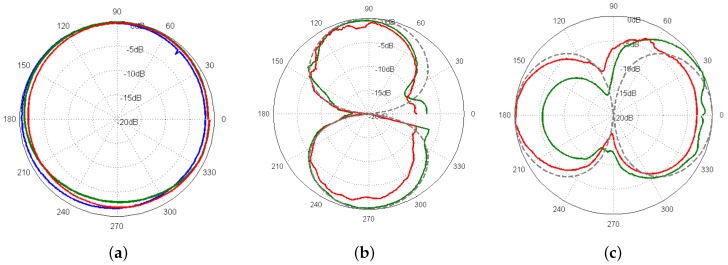
Beam patterns of the hydrophone (blue line) and accelerometer 49 (green lines) and accelerometer 50 (red lines) at 2 kHz (**a**) Polar plots the hydrophone beam pattern and the response of two accelerometers in the *x*-direction; (**b**,**c**) the beam patterns of the accelerometers in the *y* and *z* direction respectively, superimposed with the theoretical response (grey dashed line).

Figure 4 shows the directivity of the DAVS sensing elements at 2 kHz. Figure 4a shows the directivity of the hydrophone (blue curve) superimposed with the response of the two accelerometers (no 49 with green curve and no 50 with red curve) in the *x* direction. Strictly speaking, for the accelerometers, Figure 4a shows that the accelerometers are not sensitive to incident acoustic waves in the *Y-Z* plane. Quantitatively, this amounts to an error due to the cross talk of less than 3.5 dB.

Figure 4b,c show the beam patterns of the two accelerometers in the *y* and *z* direction respectively, superimposed with the theoretical (i.e., dipole) response. These results suggest that the accelerometer beam patterns have a distorted figure-of-eight shape on the plane of insonification. Theoretically, the hydrophone would be expected to be omnidirectional and the accelerometers to have a beam pattern with a figure-of-eight shape (in terms of the Directivity Index, equal to 48 dB) and 3 dB beam width of 90${}^{\circ }$. However, these results indicate that there is some asymmetry in the response of the accelerometer 49 in the *Y* direction. This can be partially attributed to a mutual interaction due to the rigidity of the structure and/or deviation of the location of the sensors in the mould from the nominal position during manufacturing. The impact of these deviations is assessed in this paper by comparing the results of in situ experiments with the GPS data estimates in Section 2.3.

### 2.3. In Situ Experiments

The device was tested on a Medusa class AUV in order to assess the device’s performance and evaluate the tolerance of the azimuth finding algorithm on the above mentioned deviations. The signal processing scheme, which was applied to the data is based on intensity measurements and does not take into account explicitly the beam pattern of the sensor elements. It assumes implicitly a perfect omnidirectional response for the hydrophone and dipole response for the accelerometer. Consequently, deviations of sensor behavior will impact on the estimation results.

**Figure 5:**
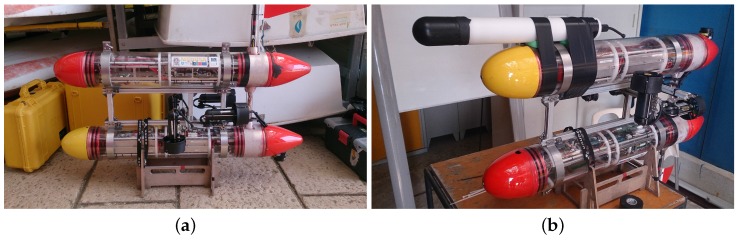
(**a**) Photo of the red MEDUSA as operated in this trial and (**b**) turned upside down position with the DAVS attached to it.

The DAVS device was tested in Lisbon at the *Oceanarium Marina* in the Parque das Nações, where the waters were protected with a sluice from current and rough sea conditions. The objective was to evaluate the ability of the DAVS to estimate the azimuthal direction of incoming sound waves when it is in motion. The DAVS was mounted on a Medusa class AUV provided by DSOR Laboratory (Instituto Superior Tecnico, IST-ID). Figure 5a shows the red MEDUSA without the DAVS and Figure 5b shows the same vehicle in inverted position with the DAVS attached to it. The AUV was sailing on the surface and was carrying a GPS antenna. It was also equipped with inertia motion sensors which register the roll, pitch and yaw of the vehicle with an update rate of 10 Hz. The GPS antenna supplied the vehicle position as an independent estimate, whilst for the data interpretation from the DAVS, the yaw data were taken into account.

**Figure 6:**
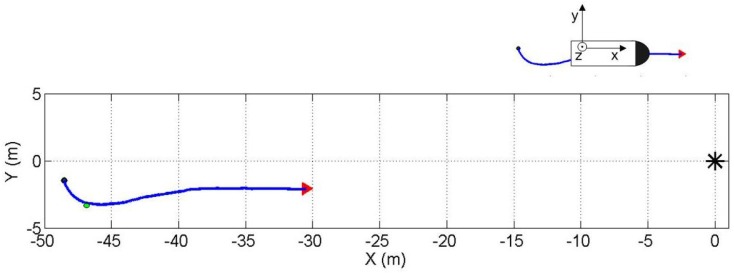
First sailed track of the Autonomous Underwater Vehicle (AUV) towards the source (black asterisk). The black dot indicates the starting point and the arrow the end point for the estimation. The illustration on the top right corner of the figure shows the sensor coordinate system with respect to the track.

**Figure 7:**
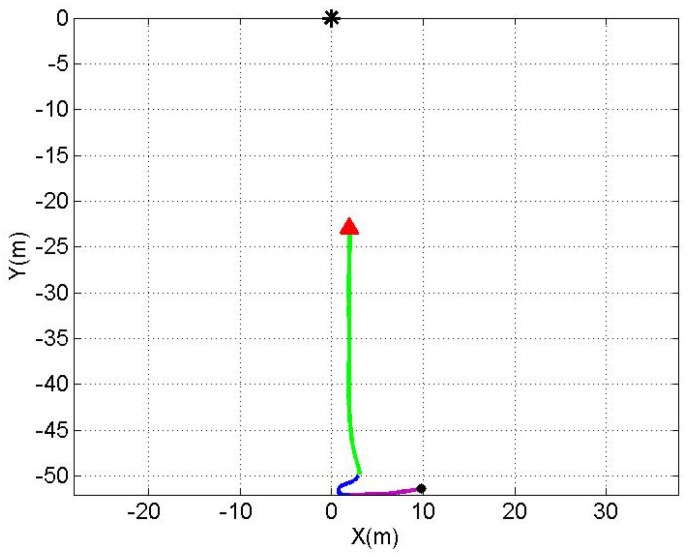
Second sailed track of the AUV towards the source (black asterisk). The black dot indicates the starting point and the arrow the end point for the estimation. The orientation of the device relative to the track is the same as the one shown in Figure 6.

The AUV was sailing on pre-programmed tracks with a nominal speed of 0.26 m/s relative to an immersed sound source (Lubell LL916C underwater speaker), which was deployed by a rope at approximately mid-water, 1.5 m depth. The depth of the DAVS during the experiment was approximately 0.5 m. Figure 6 and Figure 7 show the trajectories, which are examined in this paper. The trajectories are referenced relative to the position of the sound source (0,0) on the experimental *Y-Z* plane parallel to the sea floor. In Figure 6, the blue line shows the first trajectory for the results discussed in this paper, the black dot and the red arrow indicate the beginning and the end of the track of the acoustic data presented here. The green dot indicates the starting point for the azimuthal calculation using the AUV positional information from GPS, as an independent check for the estimates obtained with the DAVS. Figure 7 shows the second track, which is discussed in this paper. The black dot indicates the beginning of the track; the different colours for different sections of the trajectory are used for visualisation purposes and discussion of the results in Section 3.

The sound source was emitting chirp signals from 1 kHz to 2 kHz every 0.396 s. The signals were sampled at 10,547 Hz. The DAVS *x*–*y* plane was parallel to the experiment *X*–*Y* plane with the positive *z* direction pointing upwards and the positive *x* in the direction of sailing, according to the right hand coordinate convention shown in the top right of Figure 6. The DAVS was positioned on the AUV such that the two accelerometers were aligned with the vertical axis, i.e., the device’s *z* axis, with the accelerometer 50 at the top of the accelerometer 49. In this setup, two estimates were obtained from the two sets of *x* and *y* velocity components ($u_{x}\left(t\right)$ and $u_{y}\left(t\right)$ respectively) derived from the accelerometer signals and the hydrophone pressure output using:

(2) $$\hat{\Theta }=atan\frac{\langle p\left(t\right)u_{y}\left(t\right)\rangle }{\langle p\left(t\right)u_{x}\left(t\right)\rangle }$$

where $\hat{\Theta }$ is the azimuth estimate, p(t) is the pressure signal and 〈〉 denotes the time average. For the results presented in Section 3, an averaging window of 4 s was applied for each snapshot.

The sound source can be considered omnidirectional for the frequencies of the experiment (according to the specification sheet for this type of sources, the model LL916C is at 1 kHz omnidirectional and at 2 kHz omnidirectional within 1 dB). In this scenario, the azimuthal position of the source relative to the DAVS is approximated from the instantaneous angle between the tangent of the trajectory and the trajectory curve itself using Equation (2) and corrected for the yaw motion. The positional GPS sensor data were used as an independent non-acoustic position estimate.

## 3. Results

**Figure 8:**
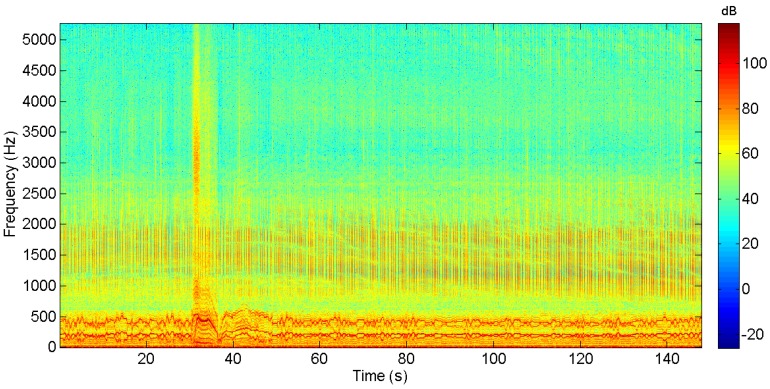
Spectrogram showing the hydrophone signal during the track of Figure 7. The data are shown in dB referenced to unity.

For each trajectory, two azimuth estimates were obtained with the DAVS using Equation (2), see reference [3] for details on the signal processing. The signals were filtered in the frequency range of the chirp signal and the estimators were computed in the time domain with an unweighed moving average filter. The azimuth estimate from the non-acoustic data was obtained from the positional information, the GPS antenna of the AUV, as mentioned in Section 2. This information was noisy and in order to compare with the acoustic estimates, the non-acoustic azimuth estimates were smoothed using a third-order Savitzky–Golay filter. Figure 8 shows a spectogram of the hydrophone signal during sailing of the second track shown in Figure 7. These results show that, for the most part of the track, the DAVS signals do not interfere with the AUV self-noise except a moment between the 30th and 35th second, at the moment the AUV initiates the sharp turn.

**Figure 9:**
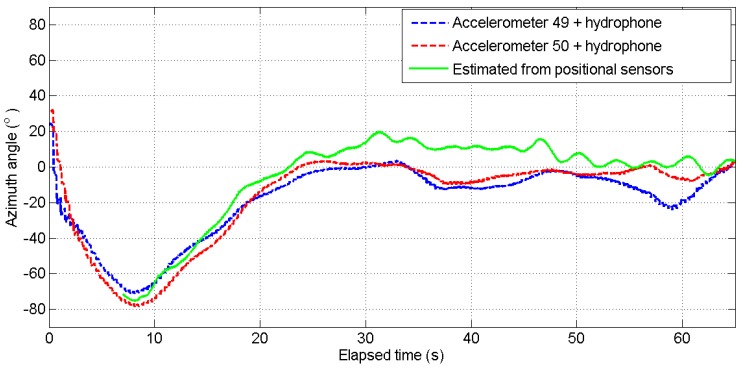
Estimation of sound azimuth direction of the sound relative to AUV sailing from the track, shown in Figure 6, as computed from the lower (red curve) and the upper (blue curve) accelerometer. The green line gives the source azimuth estimate as derived from the AUV positional sensors (GPS) starting from the point of the track indicated with the green dot in Figure 6.

**Figure 10:**
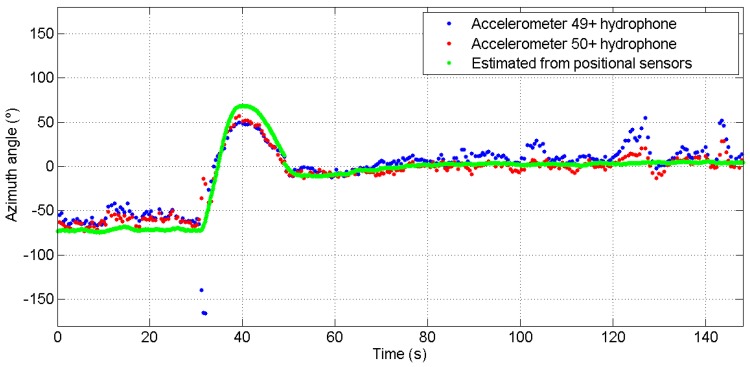
Estimation of sound azimuth direction of the sound relative to AUV sailing from the track shown in Figure 7, as computed from the lower (red curve) and the upper (blue curve) accelerometer. The green line gives the source azimuth estimate as derived from the AUV positional sensors (GPS).

Figure 9 shows the results from the dataset of the track shown in Figure 6. Two acoustic azimuth estimates (blue and red curves) are superimposed with the estimated angle from the AUV to the sound source as derived from the AUV motion sensors (green curve). One estimate is computed by combining the upper accelerometer and the hydrophone signals (blue curve) and the other estimate is obtained by combining the lower accelerometer with the same hydrophone signals (red curve). Similarly, Figure 10 shows the results from the second track examined in this paper. The time axis of this figure and Figure 8 coincide. In this figure, there are two almost constant paths, which correspond to the purple and green straight line paths of the trajectory. In the middle, the curved part corresponds to the AUV turn, i.e., the blue part of the trajectory, where it is observed that the turn is captured by the algorithm.

For both tracks, the two estimates from the two accelerometers combined with the hydrophone data show the same trend and exhibit the same motion features with those appearing at the GPS data. We observe that the change in direction is well detected but there are differences in the numerical values. Comparing the blue and red curves with the estimated angle from the track (green line), the estimates generally follow the so-called ground truth over all the tracks; numerically, an average of 20${}^{\circ }$ difference is observed. The discrepancies seen may be attributed to noise on the data or on the ground truth itself, i.e., the GPS data, since the latter is an estimate too. In addition, the discrepancy between these estimates may be attributed to two factors. One is the difference in the directivity of the two accelerometers, see Section 2.2, and another the AUV roll and pitch during sailing. The curves shown here were not corrected for the roll and pitch AUV motion as these usually introduce second-order errors in the azimuth direction.

## 4. Discussion

This paper presented azimuth estimation results from an ongoing prototype development of a Dual Accelerometer Vector Sensor. The DAVS design and construction were evaluated through testing the device’s directivity in a tank and in situ mounted on an AUV. For the latter, a simple direction finding algorithm was applied to show two independent azimuth estimates using the two accelerometers of the device. The results indicated that the angle estimation is in line with the GPS estimated track, which served as the reference signal. It was shown that for the Medusa class AUVs, there is no interference between vehicle motion and noise on the DAVS measurements. Additionally, the device is sufficiently compact to not impact on vehicle manoeuvrability, enabling it to respond to the required autonomy. Thus overall, it fulfils the initial design requirements.

The experimental results of this work suggest that without accounting for the accelerometer response difference and with no correction for the AUV motion, except for the yaw angular directions, the acoustic source could be tracked using either accelerometer. This indicates a good future potential for the DAVS, to extend its usage in acoustic surveys.

In the frequency range of interest for the experimental results discussed in this paper, the hydrophone sensitivity is within 1 dB of its design value and has an omnidirectional beam pattern within 3.5 dB. The accelerometer sensitivity is 24 mV/m/s${}^{2}$ at 2 kHz and the spatial response features a figure-of-eight beam pattern. The three sensors are moulded in the same encapsulation material and they are sufficiently acoustically decoupled to result in two azimuth estimates. Experimental results of the DAVS mounted on an AUV showed that a relatively simple algorithm was able to obtain an azimuth angle estimation of the sound source. As the device is intended for geophysical surveys with AUVs, a future improvement is the correction of the azimuth angle estimates for the overall AUV motion and investigation of other potential bias errors. The application of a higher resolution algorithm for direction finding as well as methods to minimise interference between sensors are also subject of ongoing research work.

## Figures and Tables

**Table 1:** Summary of the DAVS system design characteristics.

Characteristic	Description
Description	vector sensor with autonomous acquisition power system
	optional power and data cable
Autonomy	20 h operation with 20 V–3100 mAh battery
Bandwidth	120 Hz–4 kHz
Receiver elements	2 accelerometers and 1 Hydrophone
Accelerometers	2 PCB 356A17 accelerometers, nominal sensitivity 50 mV/m/s${}^{2}$ in air
Hydrophone	cylindrical PZT element, nominal sensitivity −195 dB re V/μ Pa
A/D converter	24 bit Sigma Delta, simultaneous sampling at 10,547 Hz or 52,734 Hz
Storage capacity	128 GB microSD card
Time synchronisation	Device RTC or host RTC when streaming, accuracy 1 s/month
Motion sensors	9 axis DoF MEMS with tri-axial accelerometer, magnetometer and gyro
Data transfer	Ethernet connection
Container dimensions	Length: 525 mm, Diameter 65 mm
Device weight	1.4 kg in air, positively buoyant in water
Maximum deployment depth	100 m
Power supply	On batteries or power cable connection to a 24 V DC
Data acquisition modes	Streaming to a computer or recorded in the device

## Abbreviations

The following abbreviations are used in this manuscript:

ADC	Analogue to Digital Converter
AUV	Autonomous Underwater Vehicles
CAD	Computer Aided Design
DAVS	Dual Accelerometer Vector Sensor
DI	Directivity index
DSOR	Dynamical Systems and Ocean Robotics Lab
DOA	Direction Of Arrival
GB	Giga Byte
GPS	Global Positioning System
IST ID	Instituto Superior Técnico—Investigação e Desenvolvimento
PGA	Programmable Gain Amplifier
PZT	Lead Zirconate Titanite
RTC	Real Time Communication
SNR	Signal to Noise Ratio
SPL	Sound Pressure Level
